# Asymptomatic HIV People Present Different Profiles of sCD14, sRAGE, DNA Damage, and Vitamins, according to the Use of cART and CD4^+^ T Cell Restoration

**DOI:** 10.1155/2018/7531718

**Published:** 2018-04-10

**Authors:** Karen Ingrid Tasca, Camila Renata Correa, Juliana Trindade Caleffi, Monica Banwart Mendes, Mariana Gatto, Vanessa Martinez Manfio, Caio Cavassan de Camargo, Francilene Capel Tavares, Mara Biasin, Lenice do Rosário de Souza

**Affiliations:** ^1^Department of Tropical Diseases, Botucatu Medical School (FMB), Universidade Estadual Paulista (UNESP), Botucatu, SP, Brazil; ^2^Department of Pathology, FMB, UNESP, Botucatu, SP, Brazil; ^3^Department of Biomedical and Clinical Sciences, University of Milan, Milan, Italy

## Abstract

We aimed to analyze markers of immune activation, inflammation, and oxidative stress in 92 asymptomatic HIV-infected patients according to the adequate (AR, >500 cells/mm^3^) or inadequate (IR, <500 cells/mm^3^) CD4^+^ T recovery and the presence or absence of antiretroviral treatment (cART). In relation to those newly diagnosed, they were divided into two groups, cART-naïve IR (nIR) and cART-naïve AR (nAR). Among those diagnosed more than five years ago, the following division was made: the cART-naïve long-term nonprogressors (LTNP); patient under cART and AR (tAR); and patients under cART and IR (tIR). We investigated the expression of soluble receptor for advanced glycation end products (sRAGE), high-mobility group-box protein −1 (HMGB1), soluble CD14 (sCD14), IL-8, IL-10, 8-isoprostane, vitamins, and DNA damage. We observed higher levels of sRAGE in tAR as compared to nIR, nAR, LTNP, and more sCD14 than in nIR and nAR. As for IL-10 levels, we found nIR > nAR > LTNP > tAR > tIR. Higher levels of 8-isoprostane were observed in nIR. LTNP presented a higher retinol dosage than tAR and less genotoxic damage induced by oxidative stress than the other groups. We suggest that the therapy, despite being related to lesser immune activation and inflammation, alters the vitamin profile and consequently increases the oxidative stress of patients. In addition, the lowest genotoxic index for LTNP indicates that both VL and cART could be responsible for the increased DNA damage. More studies are needed to understand the influence of cART on persistent immune activation and inflammation.

## 1. Introduction

HIV infects many cell types, especially CD4^+^ T lymphocytes, and its activation provokes cytopathic effects through the production of new viral copies [1]. This results into a progressive deterioration of the cellular immune system and a severe immunodepression, which makes the individual more susceptible to opportunistic diseases [2].

Until the 1990s, the most common causes of death were associated with infections caused by *Pneumocystis jirovecii*, *Toxoplasma gondii*, cytomegalovirus, and *avian-intracellulare complex mycobacteria*, among others [3]. However, with the advent of combined antiretroviral therapy (cART), the survival of HIV-infected subjects increased significantly, as a consequence of viral replication control and immunological and clinical parameter improvement [4], as well as lower rates of virus transmission [2, 5].

Despite therapeutic efficacy, some latent-infected cells keep the provirus integrated into their DNA, but without expressing viral proteins, known as “reservoirs” [6]. This condition remains until the cell is stimulated and activated, thus delaying the immune system's response against the virus and hammering the action of antiretroviral drugs [1, 6], which should be used throughout the patient's life.

It is also known that the existence of viral reservoirs contributes to intense immune activation [7], leading to a chronic inflammatory status and triggering a series of non-AIDS-associated comorbidities, such as cardiovascular, hepatic, bone, renal, and metabolic and neoplastic diseases [8–11]. In fact, these diseases are currently the leading causes of death among people living with HIV/AIDS (PLWHA) [2].

In addition to the persistent inflammation caused by HIV itself, the infection is also related to increased oxidative stress, one of the adverse effects that may be induced by therapy [12]. It is known that PLWHA, especially those under treatment, present a large imbalance between oxidants and antioxidants [13–15]. For example, in the presence of cART, decreased levels of vitamins, their precursors, and some antioxidants [16, 17] as well as high concentrations of lipid peroxidation products, such as 8-isoprostane, are observed. In addition, mitochondrial toxicity and DNA damage are also observed in this population [18, 19].

Another mechanism that participates in immune activation and inflammation is the homeostatic imbalance of the gut-associated lymphoid tissue (GALT), which promotes the rupture of the epithelial barrier and microbial translocation to the circulation, measured by lipopolysaccharide (LPS) and soluble CD14 (sCD14), among other markers [7, 20]. This immune activation, which can be triggered via Toll-like receptors (TLRs), activates the transcription factor NF*κ*b, leading to the transcription of cytokines and other inflammatory products, such as IL-6, IL-8, and HMGB1 (high-mobility group-box protein −1), and to the increase of the expression of RAGE (receptor for advanced glycation end products) and other receptors on the cell membrane [20–22]. Studies show increased inflammatory mediators in PLWHA [23], even in those under cART, which is responsible for only a partial decrease in the inflammatory status. Thus, these constant stimuli lead to immunosenescence and to the early aging process of PLWHA [8, 10, 12].

Considering the need of clarifying the mechanisms responsible for intense cellular immune activation, persistent inflammation, and oxidative stress, the aim of the present study was to investigate some of these markers in different groups of asymptomatic PLWHA, according to the use of cART and its different CD4^+^ T lymphocyte counts.

## 2. Material and Methods

### 2.1. Study Design

**Figure 1:**
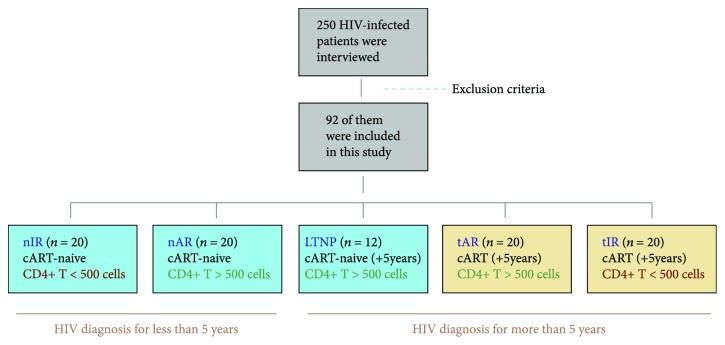
Flowchart for patients' inclusion and division into study groups, according to the presence or not of combined antiretroviral therapy (cART), CD4^+^ T cells count (cells/mm^3^), and time of HIV diagnosis.

This cross-sectional study was conducted between 2012 and 2015 at the Specialist Outpatient Service for Infectious Diseases “Domingos Alves Meira,” Botucatu Medical School Complex (FMB)-UNESP, in São Paulo state, Brazil. This service assists approximately 600 HIV-infected people from Botucatu and its surrounding area. For this study, 250 consecutive patients were interviewed, but only 94 of them were included according to the exclusion criteria. They were divided into five groups, according to Figure 1.

The intention to study groups without treatment was to investigate the influence of therapy on oxidative *status* and immune activation of PLWHA in order to open new discussions on the benefits versus harms in the early indication of cART, as these days in several countries.

### 2.2. Inclusion and Exclusion Criteria

PLWHA inclusion criteria were age between 20 and 50 years, no previous cART administration, or patients undergoing treatment for more than five years and presenting undetectable viral load (copies of HIV-1 RNA ≤50 copies/mm^3^) in such period. For them, adherence to cART was confirmed by the patient himself and by the records of medication collection at the services' pharmacy. All subjects included signed an informed consent form. Considering that many habits and comorbidities could be confounding variables and would interfere in our analysis of oxidative stress [22], patients carrying any of the following conditions were excluded: use of vitamin supplements, cancer history (current or previous), anorexia, morbid obesity, diabetes mellitus, cardiovascular, genetic or autoimmune diseases, organ transplants, use of illicit drugs and alcohol, pregnancy at any stage or breastfeeding, AIDS symptoms (those with opportunistic infections), or coinfections, such as tuberculosis or chronic viral hepatitis. For the following criteria, exclusion occurred when patients concomitantly reported two or more of them: regular performance of intense physical exercise; use of antibiotics, anxiolytics, or antidepressants; and active smoking. People with only one of these conditions were included because the statistical analysis was adjusted for these variables.

### 2.3. Sociodemographic and Clinical Data

These data were collected by interviews and from the patients' medical records, taking into account the date of blood collection for this study.

### 2.4. Analyses of Laboratory Tests

Twelve milliliters of blood was collected into an EDTA-containing tube from each patient included in the study. The material was maintained in a cooled and dark environment for 2-3 hours. After that, 60 *μ*l of total blood was separated for the comet assay procedure and the remaining sample was centrifuged at 1500 rpm for 10 minutes. Six plasma aliquots per individual were stored at −80°C until the tests were performed.

#### 2.4.1. Evaluation of Immune Activation

- *Measurement of plasma HMGB1*: a sandwich enzyme-linked immunosorbent assay (ELISA) was performed, using 100 *μ*l of a diluted sample (1 : 10) and following the manufacturer's specifications from a commercial kit (*MyBioSource*, *item MBS2707497*). Plasma HMGB1 concentration was determined by spectrophotometry, at 450 nm. Results were expressed as pg/ml using the optical density (OD) of the curves and samples for this calculation.
- *Measurement of soluble receptor for advanced glycation end products (sRAGE) and sCD14*: Using 100 *μ*l of pure samples, the sandwich ELISA protocol was developed according to the manufacturer's instructions (*R&D Systems*, *item DRG00* to sRAGE and *DC-140* to sCD14). Readings were performed immediately by a spectrophotometer at the wavelength of 450 nm. OD of the samples and curve was calculated and expressed in pg/ml.
- *Measurement of IL-8*, *and IL-10*: cytokine measurement was performed by flow cytometry (*FACSCalibur E34297502374*) using the CBA (cytometric bead array) kit of inflammatory cytokines, according to the manufacturer's instruction manual (*BD-Becton*, *Dickinson and Company*, *item551811*). The fluorescence intensity, from the cytometer, showed the amount of cytokines present in the sample. With the samples, standards containing known concentrations of each cytokine were analyzed together for the drawing of the curve, reading, and establishment of the respective detection limits. Results were obtained in pg/ml by the software *FCAP array™ (BD-Becton*, *Dickinson and Company*).

#### 2.4.2. Evaluation of Oxidative Stress

- *Evaluation of lipid peroxidation by analysis of 8 isoprostane*: The plasma aliquots destined for this assay were stored at −80°C in the presence of 0.005% BHT (butylated hydroxytoluene). ELISA was performed according to the manufacturer's instructions (*Cayman*, *item 516351*) using 50 *μ*l of plasma and read at 412 nm. Optical densities were calculated in pg/ml.
- *Analysis of DNA damage in white blood cells by the Comet Assay*: It was developed according to Sasaki et al. [24] using 30 *μ*l of whole blood set on a blade with agarose. After lysis solution treatment, triplicates of the blades were subjected to three different conditions in electrophoresis: basal condition (BAS), formamidopyrimidine DNA glycosylase (FPG), or endonuclease III (END) addition for detection of oxidative damage in purine and pyrimidine bases, respectively (*Biocompare*, *CA*, *USA*). The slides were stained by Sybr Gold (*Invitrogen*, *USA*). Using an immunofluorescence microscope connected to an image analysis system (*comet assay IV*, *Perceptive Instruments*, *Suffolk*, *Haverhill*, *UK*), a total of 50 randomly selected nucleoids were counted for each slide. Results were expressed as “tail intensity” (ti), which is the percentage of migrated DNA and “tail moment” (tm), relative values from the fraction of migrated DNA multiplied by the length of the tail.
- *Analysis of antioxidants by concentration of fat-soluble vitamins*: They were measured from 100 *μ*l of plasma by HPLC (*Waters 2996*), by a C30 column (150 × 4.6 mm, 3 *μ*m), and according to Ferreira and Matsubara [25]. The wavelength used was 455 nm for carotenoids (lutein, cryptoxanthin, lycopene and, *β*-carotene), 325 nm for retinol, and 290 nm for *α*-tocopherol. The values of the standard solution of the substances were fixed by their molar extinction coefficients expressed in *μ*mol/ml.

### 2.5. Analysis of Results

We used a generalized linear model with Poisson or negative binomial distribution for count variables and gamma distribution for asymmetric variables or binomial negative one-way ANOVA followed by Tukey-Kramer post hoc tests for those symmetric data. Pearson correlations were adopted to analyze continuous variables. After fitting the model, confounding variables (age, sex, and tobacco use, practice of intense physical activity, and use of anxiolytics and/or antidepressants) were added in order to evaluate their influence on the comparisons made. Significant differences were considered when *p* values were less than or equal to 0.05. All these procedures were performed with help from professionals at the institution's research support office using SAS for Windows, version 9.2.

This study was approved by the Research Ethics Committees of the Botucatu Medical School, registration number 4101-2011.

## 3. Results

Most of the participants were males (61.9%) aged 37 ± 8 years, white (88.1%), heterosexual (70.6%), and single (66.3%). Approximately 24% were active smokers; almost 20% practiced intense physical activity, and 4.0% used anxiolytics or antidepressants (Table 1). The groups were homogeneous as regards the above-mentioned factors (*p* > 0.05). The mean time of HIV infection, from the HIV diagnosis, was 7 ± 2 years. The lowest nadir of CD4^+^ T cells was observed in tIR, followed by tAR, and then by nIR. The means of CD4^+^ T, CD8^+^ T cells, VL, time of therapy use, and cART schemes are shown in Table 1.

**Figure 2:**
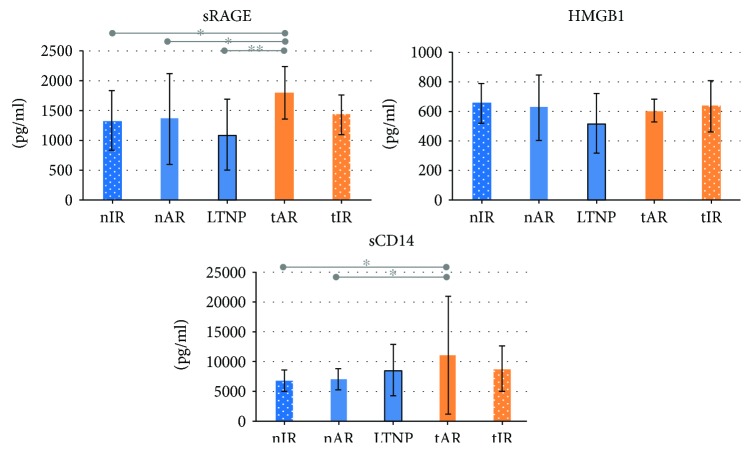
Plasma levels of soluble receptor for advanced glycation end products (sRAGE), high-mobility group-box 1 protein (HMGB1), and soluble CD14 (sCD14) proteins of the 92 people living with HIV/AIDS, according to the five studied groups. It is noted that sRAGE and sCD14 were higher in tAR than in naïve groups. Statistical tests: Tukey-Kramer for sRAGE and gamma distribution for the others. ^∗^ *p* < 0.05; ^∗∗^ *p* < 0.005.

Among the markers studied, sRAGE was higher in tAR (1798.33 ± 437.21 pg/ml) than in the naïve groups (1332.44 ± 505.94 in nIR, *p* = 0.040; 1358.67 ± 758.87 in nAR, *p* = 0.050; 1089.32 ± 600.62 pg/ml in LTNP, *p* = 0.009). tAR (11078.54 ± 9975.14 pg/ml) also showed the highest levels of sCD14; though these, differences were statistically significant only in comparison to nIR (6751.66 ± 1693.36, *p* = 0.016) and nAR (6903.02 ± 1819.33 pg/ml, *p* = 0.046). HMGB1 protein levels were not different between groups. These data are shown in Figure 2.

**Figure 3:**
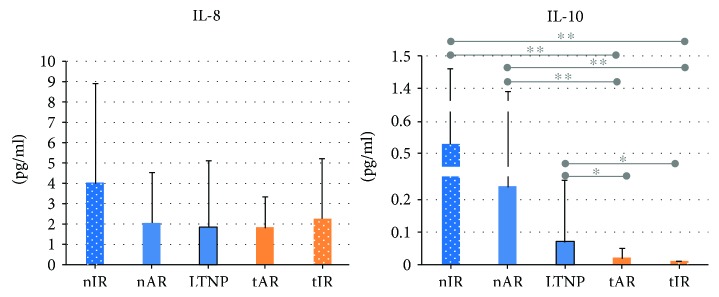
Plasma levels of IL-8 and IL-10 cytokines of the 92 people living with HIV/AIDS, according to the five study groups. It is observed that IL-10 expression was lower in the LTNP and cART groups. Statistical tests: gamma distribution. ^∗^ *p* < 0.05; ^∗∗^ *p* < 0.001.

As for cytokines, IL-8 production showed no differences among groups. Conversely, IL-10 expression was lower in the cART groups, and higher in nIR and nAR. The means of IL-10 were 0.63 ± 0.93 pg/ml in nIR, 0.24 ± 1.15 in nAR, 0.07 ± 0.19 in LTNP, 0.02 ± 0.03 in tAR, and 0.01 ± 0.00 pg/ml in tIR. Differences between naïve groups and those under cART reached statistical significance as shown in Figure 3.

**Figure 4:**
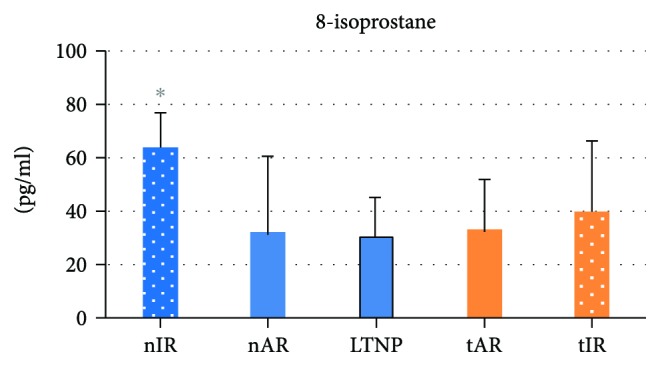
Plasma levels of 8-isoprostane of 92 people living with HIV/AIDS, according to the five composed groups. This marker is upregulated in nIR. Statistical tests: gamma distribution. ^∗^ *p* < 0.05, difference among all the other groups.

Some oxidative stress parameters were also studied, including 8-isoprostane, vitamins, and DNA damage analysis. 8-isoprostane was higher in nIR, in comparison to the other groups. As shown in Figure 4, the means and standard deviations of 8-isoprostane were 64334.88 ± 12669.83 for nIR; 31855.79 ± 28720.15 for nAR; 30367.25 ± 15091.16 for LTNP (*p* = 0.005); 32936.75 ± 18513.95 for tAR and 40018.33 ± 25888.53 for tIR (*p* < 0.050).

As for the carotenoids, the dosage of lutein was lower in tIR (5.99 ± 3.12 *μ*mol/ml) than in the other groups (in nIR, 11.47 ± 7.12, *p* = 0.006; in nAR, 12.98 ± 6.66, *p* = 0.022; in LTNP, 16.08 ± 9.00, *p* = 0.050; and in tAR, 11.76 ± 6.09 *μ*mol/ml, *p* = 0.020). There was also a difference in cryptoxanthin concentration, with nAR (16.45 ± 6.39 *μ*mol/ml, *p* = 0.003) and LTNP (18.73 ± 8.00 *μ*mol/ml, *p* = 0.041) having higher levels than the cART groups (tAR, 7.44 ± 3.88 and tIR, 7.33 ± 2.04 *μ*mol/ml) and than nIR (10.08 ± 5.55 *μ*mol/ml).

**Figure 5:**
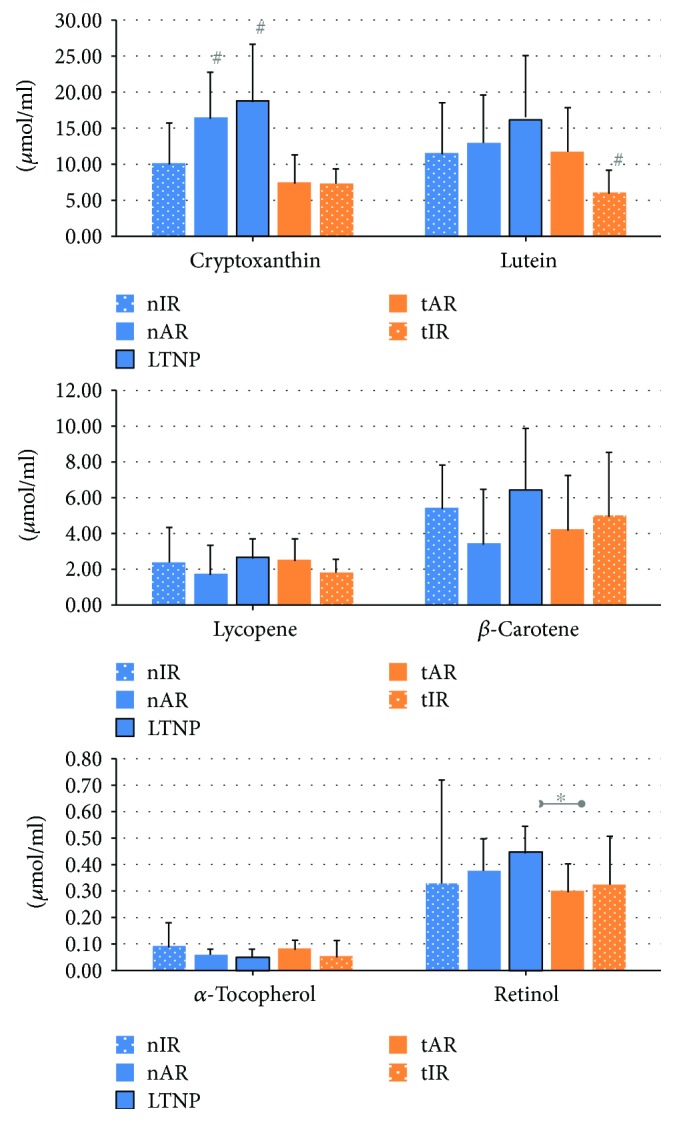
Plasma levels of carotenoids, retinol, and *α*-tocopherol of the 92 people living with HIV/AIDS, according to the five study groups. LTNP presented higher cryptoxanthin and retinol in relation to cART groups. Statistical tests: gamma distribution. ^∗^ *p* < 0.05, comparing the indicated groups; ^#^ *p* < 0.05, difference among all the other groups.

No differences were observed among the groups for *β*-carotene and lycopene dosages. Differences in mean retinol concentration were observed only between LTNP and tAR (0.45 ± 010 and 0.30 ± 0.10 *μ*mol/ml, respectively, *p* = 0.035), while *α*-tocopherol dosages were comparable among the groups included in the study (Figure 5).

**Figure 6:**
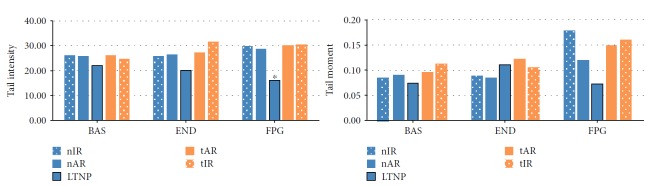
Leukocyte DNA damage of the 92 people living with HIV/AIDS, according to the five study groups, in three conditions—basal (BAS), that is, blades without enzymes treatment, or blades with enzymatic treatment (endonuclease III enzyme [END] and formanodipirimidina-DNA glycosylase, [FPG]). The LTNP group presented the lowest DNA damage; Statistical tests: gamma distribution for BAS-tm, END-tm and FPG-tm, and ANOVA for the others. ^∗^ *p* < 0.05, difference among all the other groups.

**Figure 7:**
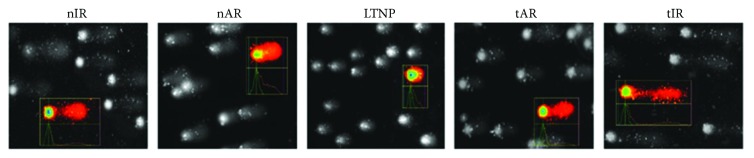
Leukocyte DNA damage representation of five selected nucleoids of patients living with HIV/AIDS, according to the five study groups, whose slides were treated with the formanodipirimidina-DNA glycosylase (FPG) enzyme. By the analysis of the Comet assay, the immunofluorescence microscopy shows that LTNP presents less damage than the other groups.

When comparing the DNA damage of the studied groups, LTNP (0.07 ± 0.02, *tail moment*) showed minor damages than the other groups, considering the slides treated with FPG (nIR, 0.18 ± 0.06, *p* = 0.033; nAR, 0.12 ± 0.08, *p* = 0.050; tAR, 0.15 ± 0.02, *p* = 0.041; and tIR, 0.16 ± 0.08, *p* = 0.035). No differences were observed among the groups for untreated (BAS) blades or those treated with END. These data are shown in Figures 6 and 7.

By Pearson analysis, positive correlations were found between CV and two markers, HMGB1 (*p* = 0.0211) and IL-10 (*p* = 0.003). Negative correlations occurred between CD4^+^ T cells and two variables, IL-8 (*p* = 0.027) and HMGB1 (*p* = 0.0240).

In addition, we verified the correlation between the markers of immune activation and some parameters that could also be related to its increase, such as CD4^+^ T *nadir*, time of HIV diagnosis, and time of therapy, and no associations were found, considering all subjects in the study together.

## 4. Discussion

The recent introduction of cART correlates with both longer life expectancy of PLWHA and the development of non-AIDS comorbidities, which occur earlier in HIV-infected subjects than in the general population [10–12]. The “early aging” of these subjects is caused by constant immune activation and chronic inflammation which leads to the exhaustion of the immune system and the imbalance of cytokines and other immunological and physiological components [8, 10, 12].

Several receptors and proteins promote cellular activation and consequently the activation of the signaling cascade that give rise to the inflammatory components. RAGE is a pattern recognition receptor (PRR) which activates the cell due to its interaction with its ligands, for example, AGEs and HMGB1 [25]. Conversely, its soluble form, sRAGE [26], acts as a suppressor of activation, since it arrests the RAGE ligands, preventing the interaction between them and the subsequent cellular signaling [22].

We detected higher sRAGE levels in the cART group with higher CD4^+^ T cell counts as compared to the non-cART groups. The possible explanation for this observation is more than a few. First, in the blood circulation of these individuals, there could be a greater accumulation of ligands for this receptor [22], such as sCD14, which was increased in the tAR group in the present study. Second, the levels of sRAGE could mirror the possible partial decrease of inflammation presented by cART patients [23]. This last argument could be justified, even, by the smaller dosages of IL-8 found in the same group of patients, although differences did not reach statistical significance. High concentrations of sRAGE have been related to the fewer occurrences of atherosclerosis in PLWHA [27], hypercholesterolemia [28], and arterial hypertension [29] in individuals not infected by HIV, suggesting that its dosage could be a useful tool in the diagnosis of cardiovascular diseases in PLWHA [27].

HMGB1 in its extracellular form is secreted actively following cellular stimulus, or passively by necrotic and apoptotic cells, and performs similar functions to those of proinflammatory cytokines [30]. Due to its binding to RAGE and other PRRs present in CD4^+^ T cells, HMGB1 may even induce HIV reactivation [31]. We did not find any difference in the levels of this protein among the five groups. However, HMGB1 correlated negatively with CD4^+^ T counts and positively with VL, validating the longitudinal study by Trøseid et al. [32], who showed a significant HMGB1 reduction after introduction of cART.

Youn et al. [21] found that monocyte stimulation with the association of LPS and HMGB1 led to higher TNF-*α* production compared to LPS alone. Taken together, the findings by Trøseid et al. [32] and Youn et al. [21] may indicate that higher levels of HMGB1 might contribute to poorer clinical outcomes in HIV-infected individuals, as there would probably be more cellular activation, reactivation of the latent virus, and increased production of inflammatory cytokines in these individuals. In addition, elevated HMGB1 plasma levels are related to other chronic inflammatory conditions in non-HIV-infected individuals including diabetes mellitus and cancer [25, 33]. Thus, there is a need to investigate the influence of cART on the decrease of this marker in order to delay the development of HIV infection and the appearance of non-AIDS comorbidities.

Among microbial translocation markers, sCD14 is related to monocyte activation and its concentration is higher in PLWHA compared to uninfected individuals. It is also associated with the characteristic comorbidities of aging in this population [34, 35]. Here, the group of individuals receiving cART and presenting high CD4^+^ T cell counts showed higher levels of sCD14 as compared to naïve groups and to those with recent infection, which was also demonstrated by Sandler et al. [36]. This fact could be related to the time of infection and therapy because, despite viral suppression at plasma levels, GALT is one of the viral reservoirs that can sustain HIV replication [37] in a T cell-deficient environment, whereas cART is not able to completely restore Th17 cells in GALT [38]. However, in the present study, the increase in sCD14 did not occur in cART patients presenting CD4^+^ T cells below 500 cells/mm^3^, as it would be expected, since these individuals would probably have fewer T lymphocytes and an even scarcer immune response in GALT, which would further compromise the balance of this mucosa.

Differently from our results, other authors have shown a relationship between greater microbial/polymicrobial translocation in immunological nonresponders and associated it with intestinal flora imbalance [39]. The importance of studying the sCD14 marker in PLWHA is also justified by the observations that there is an association between sCD14 and increased risk for cardiovascular disease [40] and that sCD14 is a predictor of all mortality causes in HIV patients, even in those with undetectable VL [36].

In HIV infection, there is imbalance not only of the Th17 profile but also of Th1, Th2, and regulatory T cells (Treg). Additionally, large numbers of inflammatory cytokines are found in HIV-positive patients, which may influence the development to AIDS and the onset of non-AIDS comorbidities [41]. IL-10 is a regulator of the inflammatory immune response [42]. In the present study, this cytokine showed different concentrations in the different groups. The highest levels were found in naïve patients presenting a recent infection, intermediate levels in naïve patients who had been diagnosed more than five years before, and the lowest in individuals under cART. These results agree with those by Brockman et al. [42], which showed a reduction in both IL-10 plasma levels and its mRNA expression in cART subjects presenting adequate viral suppression as compared to naïve individuals, with the exception of elite controls, who presented similar values to those of uninfected individuals, suggesting that viral replication may be the main determinant of cytokine concentrations. Likewise, in the present study, a positive correlation was also found between VL and IL-10 levels.

As reported by Haissman et al. [43], IL-8 showed a negative correlation with CD4^+^ T cells, which evidenced higher levels of inflammatory cytokines in individuals with CD4^+^ T cell counts below 200 cells/mm^3^. Thus, the monitoring of inflammatory cytokines could be included in the follow-up of patients in order to evaluate the evolution of HIV infection.

It is known that the constant presence of these inflammatory components and the residual replication of HIV induce oxidative stress in PLWHA, which occurs when there is overproduction of ROS and RNS or a reduction in antioxidant capacity. Such oxidative stress is potentiated by the toxic effects of cART [19]. For example, PIs are known to deregulate ubiquitin-proteasome proteins (UPS) that will contribute to endoplasmic reticulum stress, as well as to lipid accumulation, the development of insulin resistance and diabetes mellitus, and an increased risk for atherosclerosis [44]. In addition, PIs activate intracellular apoptosis pathways and increase the prooxidant status of the intracellular environment [44]. On the other hand, NRTIs inhibit DNA polymerase, decreasing mitochondrial DNA and presenting membrane loss, lower respiratory rates, and oxidative phosphorylation, which consequently induces greater production of ROS and oxidative stress [45].

We found that 8-isoprostane, a marker of lipid peroxidation, was higher in naïve individuals with CD4^+^ T cells below 500 cells/mm^3^ than in the other groups. This can be explained by the association between high oxidative stress in patients who have high VL and a poor immune system. Thus, in our study, cART does not appear to have increased levels of this marker, differently from other findings by Redhage et al. [18] and Hulgan et al. [14] in which cART individuals, even those with a controlled viral replication, had increased levels of 8-isoprostane. These authors also pointed out that PLWHA under cART without NNRTI in their composition had higher rates of 8-isoprostane as compared to those who used it or to naïve individuals. High levels of this marker are also found in several non-AIDS comorbidities, including atherosclerosis [46], which highlights the need for further studies aimed at reducing this parameter in PLWHA.

Carotenoids have the ability to arrest free radicals, and many are precursors of retinol or vitamin A. In the immune system, retinol stimulates phagocytosis, T cell proliferation, activation of cell-mediated cytotoxicity, and antibody production and contributes to intestinal mucosal homeostasis [47, 48]. However, studies have shown deficiency of *β*-carotene and retinol in the HIV-infected population [49, 50] as compared to uninfected individuals, which was also evident in subjects under cART [49]. As for the carotenoids and the dosages of *β*-carotene and lycopene, the groups showed no difference between each other. However, the lutein level was lower in the cART group presenting low CD4^+^ T cell counts, suggesting that both the use of cART and immunodeficiency could influence lutein levels. Cryptoxanthin also appears to be influenced by cART and the immune response. Indeed, its concentration increased in naïve patients with high levels of CD4^+^ T cells compared to naïve individuals with an inadequate immune response or the cART groups. Retinol was also higher in naïve patients with high CD4^+^ T cell counts, proving the importance of the natural mechanisms of HIV infection control that these individuals show.

*α*-Tocopherol is a lipid-soluble antioxidant that acts by blocking the lipid peroxidation of polyunsaturated fatty acids from membranes and lipoproteins. It also blocks the activation of NF*κ*B and the consequent production of proinflammatory cytokines and has physiological potential in the reduction of atherosclerosis [51]. There was no difference in the *α*-tocopherol levels between the groups studied here, but for other authors, there was a decrease in vitamin E [52] in individuals with low CD4^+^ T cell count. Such differences may be related to different study designs and characteristics of the population. However, we did not evaluate the cART schemes used by the participants or their vitamin E percent deficiency. In a Brazilian study, deficiency of this vitamin was found in almost 20% of PLWHA, which occurred more frequently in cART patients who did not use NNRTI in their composition [17]. Following the antioxidant deficiency in this population and their importance in reducing oxidative stress, their monitoring should be routinely introduced and micronutrient supplements should be recommended when necessary.

In the long term, oxidative stress leads to genotoxic effects which can either be repaired or lead to mutagenicity [53]. When comparing DNA damage in our groups, there was less oxidative damage in naïve patients with infection for more than five years and CD4^+^ T cell counts higher than 500 cells/mm^3^. One possible explanation is that as these individuals have good control of viral replication and CD4^+^ T counts without the cART administration, they might be able to activate more efficient DNA repair mechanisms [54]. After all, it is known that both HIV VL and antiretrovirals may contribute to genotoxic increase in these patients [14, 55, 56], as observed in the other groups studied here.

There are few human studies on the frequency of DNA damage and its consequences for HIV infection and the appearance of other comorbidities, and there are few *in vivo* studies about the interference of cART in this context. Considering that genomic instability may contribute to the development of neoplasias and that such comorbidities are common in the HIV-infected population, it would be interesting to have more studies evaluating the influence of chronic use of cART as well as the persistent immune activation and inflammation in PLWHA in these genotoxic alterations.

This study present some limitations, such as its cross-sectional design, reduced number of individuals in LTNP group, and the lack of food surveys or anthropometric measures. However, other factors were deeply considered, such as strict exclusion criteria, group homogeneity regarding sociodemographic variables, and data analysis adjusted for gender, age, tobacco use, intense physical activity, and use of anxiolytics and/or antidepressants.

## 5. Conclusions

We found that patients with cART, viral suppression for over five years, and high CD4^+^ T cell count (> 500 cells/mm^3^) have higher levels of sRAGE and sCD14 and this group along with that under cART and low CD4^+^ T cell count showed lower levels of IL-10 and vitamins compared to naïve subjects. This result suggests that cART may not restore cell functions in GALT, which would compromise local homeostasis, induce microbial translocation, and consequently lead to the increase of some soluble ligands (e.g., sRAGE) in an attempt to minimize the activation, via RAGE or TLR. In addition, despite the incontestable benefits of cART, these drugs can influence the antioxidant defense of the body, considerably reducing some vitamins which could compromise the oxidative balance and contribute to the persistent inflammation in PLWHA.

We also showed that high plasma concentrations of 8-isoprostane occurred in naïve individuals with CD4^+^ T cells below 500 cells/mm^3^, evidencing the participation of HIV viral replication in increasing oxidative stress. Regarding DNA damage, the group of naïve patients with CD4^+^ T cell count higher than 500 cells/mm^3^ and diagnosed more than five years ago (LTNP) showed a lower genotoxic index than all the other groups, indicating that both VL and cART may be responsible for the DNA damage increase, a process that could perhaps be alleviated by specific intrinsic factors of the organism, such as more efficient repair mechanisms and protective genes in certain individuals.

Further studies are needed to understand the persistent mechanisms of activation and inflammation, influenced or not by cART, in order to guarantee greater longevity and better quality of life for PLWHA.

## Figures and Tables

**Table 1:** Epidemiological and clinical characterization of 92 HIV-infected individuals studied.

Variables	nIR^(1)^	nAR^(2)^	LTNP^(3)^	tAR^(4)^	tIR^(5)^	*p* value
Age (ME ± SD; years)	34.0 ± 8.3	36.9 ± 9.4	35.8 ± 6.2	39.1 ± 7.1	40.0 ± 7.1	*0.057*
Males (*n*/%)	14/70.0%	13/65.0%	5/41.6%	10/50.0%	13/65.0%	*0.288*
Heterosexual (*n*/%)	12/60.0%	14/70.0%	10/83.3%	15/75.0%	14/70.0%	*0.600*
Single (*n*/%)	14/70.0%	13/65.0%	7/58.3%	11/55.0%	15/75.0%	*0.695*

Smokers (*n*/%)	7/35.0%	3/15.0%	4/33.3%	3/15.0%	4/20.0%	*0.531*
Physical activity (*n*/%)	2/10.0%	5/25.0%	2/16.6%	6/30.0%	3/15.0%	*0.304*
Antidepressants (*n*/%)	2/10.0%	1/5.0%	0/0.0%	0/0.0%	1/5.0%	*0.859*

CD4^+^ T (ME ± SD; cells)	341.6 ± 121.9^∗^^(2–4)^	699.3 ± 142.8	862.1 ± 360.1	833.7 ± 212.5	343.3 ± 140.0^∗^^(2–4)^	*<0.001*
CD8^+^ T (ME ± SD; cells)	1120.2 ± 903.0	1431.1 ± 739.9^∗^^(4, 5)^	1261.3 ± 672.6	899.7 ± 292.6	880.1 ± 579.9	*<0.010*
*Nadir* CD4 (ME ± SD; cells)	316.5 ± 143.7^∗^^(2)^	568.7 ± 122.9	526.5 ± 244.5	294.4 ± 162.2^∗^^(1, 2)^	98.1 ± 92.4^∗^^all^	*<0.010*
VL (ME ± SD; RNA copies)	527 ± 1394^∗^^(2, 3)^	67 ± 58	36 ± 79	Undetectable	Undetectable	*<0.001*
HIV diagnosis (ME ± SD; years)	2.1 ± 1.0^∗^^(4, 5)^	1.9 ± 0.8^∗^^(4, 5)^	7.6 ± 2.2	14.5 ± 3.9	10.6 ± 4.3	*<0.010*

Time of cART (ME ± SD; years)	—	—	—	9.0 ± 4.4	8.3 ± 3.5	*ne*
PI-based scheme (*n*/%)	—	—	—	13/32.5%	10/25.0%	*0.587*
NNRTI-based scheme (*n*/%)	—	—	—	7/17.5%	8/20.0%	*0.500*
Rescue therapy (*n*/%)	—	—	—	0/0.0%	2/5.0%	*0.269*

cART: combined antiretroviral therapy; ne: not evaluated; ME: mean; SD: standard deviation; *n*: number of subjects; VL-HIV plasma viral load (expressed in copies × 10^3^/ml); CD4^+^ T and CD8^+^ T cell counts (expressed in number of cells/mm^3^); nIR, 20 cART-naïve with CD4^+^ T < 500 cells/mm^3^; nAR, 20 naïve with CD4^+^ T > 500; LTNP, 12 naïve with CD4^+^ T > 500 and infection for more than five years; tAR, 20 cART patients with undetectable VL and CD4^+^ T > 500 for more than five years; tIR, 20 cART patients with undetectable VL and CD4^+^ T < 500 for more than 5 years; IP: protease inhibitor; NNRTI: nonnucleoside reverse transcriptase inhibitor. Statistical tests: negative binomial, ANOVA, Poisson, and Chi-square. ^∗^Statistical differences between specified groups (1–5).

## Supplementary Materials

Supplementary Materials Table of summary data of the various biomarkers studied in 94 PLWHA.

## References

[1] Alexaki A., Liu Y., Wigdahl B. (2008). Cellular reservoirs of HIV-1 and their role in viral persistence. *Current HIV Research*.

[2] UNAIDS *Joint United Nations Programme on HIV/AIDS. Global AIDS Response Progress Reporting*.

[3] Hirschtick R. E., Glassroth J., Jordan M. C. (1995). Bacterial pneumonia in persons infected with the human immunodeficiency virus. *New England Journal of Medicine*.

[4] Ministério da Saúde (2013). *Novo Protocolo Clinico e Diretrizes Terapêuticas para Manejo da Infecção pelo HIV em Adultos*.

[5] Cohen M. S., Chen Y. Q., McCauley M. (2011). Prevention of HIV-1 infection with early antiretroviral therapy. *New England Journal of Medicine*.

[6] Pantaleo G., Graziosi C., Butini L. (1991). Lymphoid organs function as major reservoirs for human immunodeficiency virus. *Proceedings of the National Academy of Sciences of the United States of America*.

[7] Appay V., Sauce D. (2008). Immune activation and inflammation in HIV-1 infection: causes and consequences. *The Journal of Pathology*.

[8] Hsu D. C., Sereti I., Ananworanich J. (2013). Serious non-AIDS events: immunopathogenesis and interventional strategies. *AIDS Research and Therapy*.

[9] Cowell A., Shenoi S. V., Kyriakides T. C., Friedland G., Barakat L. A. (2015). Trends in hospital deaths among human immunodeficiency virus–infected patients during the antiretroviral therapy era, 1995 to 2011. *Journal of Hospital Medicine*.

[10] Önen N. F., Overton E. T., Seyfried W. (2010). Aging and HIV infection: a comparison between older HIV-infected persons and the general population. *HIV Clinical Trials*.

[11] Deeks S. G., Phillips A. N. (2009). HIV infection, antiretroviral treatment, ageing, and non-AIDS related morbidity. *BMJ*.

[12] Smith R. L., de Boer R., Brul S., Budovskaya Y., van der Spek H. (2013). Premature and accelerated aging: HIV or HAART?. *Frontiers in Genetics*.

[13] Ngondi J. L., Oben J., Etame L. H., Forkah D. M., Mbanya D. (2006). The effect of different combination therapies on oxidative stress markers in HIV infected patients in Cameroon. *AIDS Research and Therapy*.

[14] Hulgan T., Morrow J., D'Aquila R. T. (2003). Oxidant stress is increased during treatment of human immunodeficiency virus infection. *Clinical Infectious Diseases*.

[15] Mandas A., Iorio E. L., Congiu M. G. (2009). Oxidative imbalance in HIV-1 infected patients treated with antiretroviral therapy. *Journal of Biomedicine and Biotechnology*.

[16] Suresh D. R., Annam V., Pratibha K., Maruti Prasad B. V. (2009). Total antioxidant capacity – a novel early bio-chemical marker of oxidative stress in HIV infected individuals. *Journal of Biomedical Science*.

[17] Itinoseki Kaio D. J., Rondó P. H. C., Luzia L. A., Souza J. M. P., Firmino A. V., Santos S. S. (2014). Vitamin E concentrations in adults with HIV/AIDS on highly active antiretroviral therapy. *Nutrients*.

[18] Redhage L. A., Shintani A., Haas D. W. (2009). Clinical factors associated with plasma F_2_-isoprostane levels in HIV-infected adults. *HIV Clinical Trials*.

[19] Blas-García A., Apostolova N., Esplugues J. V. (2011). Oxidative stress and mitochondrial impairment after treatment with anti-HIV drugs: clinical implications. *Current Pharmaceutical Design*.

[20] Brenchley J. M., Price D. A., Schacker T. W. (2006). Microbial translocation is a cause of systemic immune activation in chronic HIV infection. *Nature Medicine*.

[21] Youn J. H., Oh Y. J., Kim E. S., Choi J. E., Shin J. S. (2008). High mobility group box 1 protein binding to lipopolysaccharide facilitates transfer of lipopolysaccharide to CD14 and enhances lipopolysaccharide-mediated TNF-*α* production in human monocytes. *The Journal of Immunology*.

[22] Kalea A. Z., Schmidt A. M., Hudson B. I. (2009). RAGE: a novel biological and genetic marker for vascular disease. *Clinical Science*.

[23] French M. A., King M. S., Tschampa J. M., da Silva B. A., Landay A. L. (2009). Serum immune activation markers are persistently increased in patients with HIV infection after 6 years of antiretroviral therapy despite suppression of viral replication and reconstitution of CD4^+^ T cells. *The Journal of Infectious Diseases*.

[24] Sasaki Y. F., Kawaguchi S., Kamaya A. (2002). The comet assay with 8 mouse organs: results with 39 currently used food additives. *Mutation Research/Genetic Toxicology and Environmental Mutagenesis*.

[25] Sims G. P., Rowe D. C., Rietdijk S. T., Herbst R., Coyle A. J. (2010). HMGB1 and RAGE in inflammation and cancer. *Annual Review of Immunology*.

[26] Schmidt A. M., Yan S. D., Yan S. F., Stern D. M. (2001). The multiligand receptor RAGE as a progression factor amplifying immune and inflammatory responses. *Journal of Clinical Investigation*.

[27] Jeong S. J., Kim C. O., Song Y. G. (2011). Low plasma levels of the soluble receptor for advanced glycation end products in HIV-infected patients with subclinical carotid atherosclerosis receiving combined antiretroviral therapy. *Atherosclerosis*.

[28] Santilli F., Bucciarelli L., Noto D. (2007). Decreased plasma soluble RAGE in patients with hypercholesterolemia: effects of statins. *Free Radical Biology & Medicine*.

[29] Geroldi D., Falcone C., Emanuele E. (2005). Decreased plasma levels of soluble receptor for advanced glycation end-products in patients with essential hypertension. *Journal of Hypertension*.

[30] Scaffidi P., Misteli T., Bianchi M. E. (2002). Release of chromatin protein HMGB1 by necrotic cells triggers inflammation. *Nature*.

[31] Thierry S., Gozlan J., Jaulmes A. (2007). High-mobility group box 1 protein induces HIV-1 expression from persistently infected cells. *AIDS*.

[32] Trøseid M., Sönnerborg A., Nowak P. (2011). High mobility group box protein-1 in HIV-1 infection: connecting microbial translocation, cell death and immune activation. *Current HIV Research*.

[33] Zhang S., Zhong J., Yang P., Gong F., Wang C. Y. (2009). HMGB1, an innate alarmin, in the pathogenesis of type 1 diabetes. *International Journal of Clinical and Experimental Pathology*.

[34] Schouten J., Wit F. W., Stolte I. G. (2014). Cross-sectional comparison of the prevalence of age-associated comorbidities and their risk factors between HIV-infected and uninfected individuals: the AGEhIV cohort study. *Clinical Infectious Diseases*.

[35] Klatt N. R., Funderburg N. T., Brenchley J. M. (2013). Microbial translocation, immune activation, and HIV disease. *Trends in Microbiology*.

[36] Sandler N. G., Wand H., Roque A. (2011). Plasma levels of soluble CD14 independently predict mortality in HIV infection. *The Journal of Infectious Diseases*.

[37] d'Ettorre G., Paiardini M., Zaffiri L. (2011). HIV persistence in the gut mucosa of HIV-infected subjects undergoing antiretroviral therapy correlates with immune activation and increased levels of LPS. *Current HIV Research*.

[38] Bixler S. L., Mattapallil J. J. (2013). Loss and dysregulation of Th17 cells during HIV infection. *Clinical and Developmental Immunology*.

[39] Merlini E., Bai F., Bellistrì G. M., Tincati C., d'Arminio Monforte A., Marchetti G. (2011). Evidence for polymicrobic flora translocating in peripheral blood of HIV-infected patients with poor immune response to antiretroviral therapy. *PLoS One*.

[40] Merlini E., Luzi K., Suardi E. (2012). T-cell phenotypes, apoptosis and inflammation in HIV+ patients on virologically effective cART with early atherosclerosis. *PLoS One*.

[41] Tasca K. I., Calvi S. A., do Rosário de Souza L. (2012). Immunovirological parameters and cytokines in HIV infection. *Revista da Sociedade Brasileira de Medicina Tropical*.

[42] Brockman M. A., Kwon D. S., Tighe D. P. (2009). IL-10 is up-regulated in multiple cell types during viremic HIV infection and reversibly inhibits virus-specific T cells. *Blood*.

[43] Haissman J. M., Vestergaard L. S., Sembuche S. (2009). Plasma cytokine levels in Tanzanian HIV-1-infected adults and the effect of antiretroviral treatment. *JAIDS Journal of Acquired Immune Deficiency Syndromes*.

[44] Reyskens K. M. S. E., Essop M. F. (2014). HIV protease inhibitors and onset of cardiovascular diseases: a central role for oxidative stress and desregulation of the ubiquitin–proteasome system. *Biochimica et Biophysica Acta (BBA) - Molecular Basis of Disease*.

[45] Nolan D., Mallal S. (2004). Complications associated with NRTI therapy: update on clinical features and possible pathogenic mechanisms. *Antiviral Therapy*.

[46] Shishehbor M. H., Zhang R., Medina H. (2006). Systemic elevations of free radical oxidation products of arachidonic acid are associated with angiographic evidence of coronary artery disease. *Free Radical Biology & Medicine*.

[47] de Souza W. A., da Costa Vilas Boas O. M. G. (2002). A deficiência de vitamina A no Brasil: um panorama. *Revista Panamericana de Salud Pública*.

[48] Mora J. R., Iwata M., von Andrian U. H. (2008). Vitamin effects on the immune system: vitamins a and D take centre stage. *Nature Reviews Immunology*.

[49] Kaio D. J., Rondó P. H. C., Souza J. M. P., Firmino A. V., Luzia L. A., Segurado A. A. (2013). Vitamin A and beta-carotene concentrations in adults with HIV/AIDS on highly active antiretroviral therapy. *Journal of Nutritional Science and Vitaminology*.

[50] Baeten J. M., Mcclelland R. S., Wener M. H. (2007). Relationship between markers of HIV-1 disease progression and serum *β*-carotene concentrations in Kenyan women. *International Journal of STD & AIDS*.

[51] Munteanu A., Zingg J. M. (2007). Cellular, molecular and clinical aspects of vitamin E on atherosclerosis prevention. *Molecular Aspects of Medicine*.

[52] Bilbis L. S., Idowu D. B., Saidu Y., Lawal M., Njoku C. H. (2010). Serum levels of antioxidant vitamins and mineral elements of human immunodeficiency virus positive subjects in Sokoto, Nigeria. *Annals of African Medicine*.

[53] Lord C. J., Ashworth A. (2012). The DNA damage response and cancer therapy. *Nature*.

[54] Peraire J., Miro O., Saumoy M. (2007). HIV-1-infected long-term non-progressors have milder mitochondrial impairment and lower mitochondrially-driven apoptosis in peripheral blood mononuclear cells than typical progressors. *Current HIV Research*.

[55] Wu K.-M., Powley M. W., Ghantous H. (2012). Timing of carcinogenicity studies and predictability of genotoxicity for tumorigenicity in anti-HIV drug development. *International Journal of Toxicology*.

[56] Nunnari G., Smith J. A., Daniel R. (2008). HIV-1 Tat and AIDS-associated cancer: targeting the cellular anti-cancer barrier?. *Journal of Experimental & Clinical Cancer Research*.

