# Supplementary material for: Asymptomatic HIV People Present Different Profiles of sCD14, sRAGE, DNA Damage, and Vitamins, according to the Use of cART and CD4+ T Cell Restoration
Source: J Immunol Res. 2018 Apr 10;2018:7531718. doi: 10.1155/2018/7531718 (PMC5914107; doi:10.1155/2018/7531718)
Supplement: Supplementary Materials — Table of summary data of the various biomarkers studied in 94 PLWHA. [file 7531718.f1.pdf]

Supplementary materials

Table of summary data of the various biomarkers studied in 94 PLWHA

	nIR	nAR	LTNP	tAR	tIR
**sRAGE**	-	-	-	> than nIR, nAR, LTNP	-
**HMGB1**	-	-	-	-	-
**sCD14**	-	-	-	> than nIR, nAR	-
**IL-8**	-	-	-	-	-
**IL-10**	> than tAR, tIR	> than tAR, tIR	-	-	-
**8-isoprostane**	> than all groups	-	-	-	-
**cryptoxanthin**	-	> than all groups	> than all groups	-	-
**lutein**	-	-	-	-	> than all groups
**lycopene**	-	-	-	-	-
**β-carotene**	-	-	-	-	-
**α-tocopherol**	-	-	-	-	-
**retinol**	-	-	> than tAR	-	-
**DNA damage**	-	-	> than all groups	-	-

Among the markers studied, sRAGE was higher in tAR than in the naïve groups. The same group (tAR) also showed the highest levels of sCD14, though these differences were statistically significative only in comparison to nIR and nAR. HMGB1 protein levels were not different between groups. As for cytokines, IL-8 production showed no differences among groups. Conversely, IL-10 expression was lower in the cART-groups, and higher in nIR and nAR. 8-isoprostane was higher in nIR, in comparison to the other groups. As for the carotenoids, the dosage of lutein was lower in tIR than in the other groups. There was also a difference in cryptoxanthin concentration, with nAR and LTNP having higher levels than the cART groups and than nIR. No differences were observed among the groups for β-carotene and lycopene dosages. Differences in mean retinol concentration were observed only between LTNP and tAR, while α-tocopherol dosages were comparable among the groups included in the study. When
